# Machine learning for subtype definition and risk prediction in heart failure, acute coronary syndromes and atrial fibrillation: systematic review of validity and clinical utility

**DOI:** 10.1186/s12916-021-01940-7

**Published:** 2021-04-06

**Authors:** Amitava Banerjee, Suliang Chen, Ghazaleh Fatemifar, Mohamad Zeina, R. Thomas Lumbers, Johanna Mielke, Simrat Gill, Dipak Kotecha, Daniel F. Freitag, Spiros Denaxas, Harry Hemingway

**Affiliations:** 1 Institute of Health Informatics, University College London, 222 Euston Road, London, NW1 2DA UK; 2 Health Data Research UK, University College London, London, UK; 3 University College London Hospitals NHS Trust, 235 Euston Road, London, UK; 4 Barts Health NHS Trust, The Royal London Hospital, Whitechapel Rd, London, UK; 5 Medical School, King’s College London, London, UK; 6 Bayer AG, Division Pharmaceuticals, Open Innovation & Digital Technologies, Wuppertal, Germany; 7 University of Birmingham Institute of Cardiovascular Sciences and University Hospitals Birmingham NHS Foundation Trust, Birmingham, UK; 8 Department of Cardiology, University Medical Centre Utrecht, Utrecht, the Netherlands; 9 The Alan Turing Institute, London, UK; 10 University College London Hospitals Biomedical Research Centre (UCLH BRC), London, UK

**Keywords:** Cardiovascular disease, Machine learning, Subtype, Risk prediction, Informatics, Systematic review

## Abstract

**Background:**

Machine learning (ML) is increasingly used in research for subtype definition and risk prediction, particularly in cardiovascular diseases. No existing ML models are routinely used for cardiovascular disease management, and their phase of clinical utility is unknown, partly due to a lack of clear criteria. We evaluated ML for subtype definition and risk prediction in heart failure (HF), acute coronary syndromes (ACS) and atrial fibrillation (AF).

**Methods:**

For ML studies of subtype definition and risk prediction, we conducted a systematic review in HF, ACS and AF, using PubMed, MEDLINE and Web of Science from January 2000 until December 2019. By adapting published criteria for diagnostic and prognostic studies, we developed a seven-domain, ML-specific checklist.

**Results:**

Of 5918 studies identified, 97 were included. Across studies for subtype definition (*n* = 40) and risk prediction (*n* = 57), there was variation in data source, population size (median 606 and median 6769), clinical setting (outpatient, inpatient, different departments), number of covariates (median 19 and median 48) and ML methods. All studies were single disease, most were North American (*n* = 61/97) and only 14 studies combined definition and risk prediction. Subtype definition and risk prediction studies respectively had limitations in development (e.g. 15.0% and 78.9% of studies related to patient benefit; 15.0% and 15.8% had low patient selection bias), validation (12.5% and 5.3% externally validated) and impact (32.5% and 91.2% improved outcome prediction; no effectiveness or cost-effectiveness evaluations).

**Conclusions:**

Studies of ML in HF, ACS and AF are limited by number and type of included covariates, ML methods, population size, country, clinical setting and focus on single diseases, not overlap or multimorbidity. Clinical utility and implementation rely on improvements in development, validation and impact, facilitated by simple checklists. We provide clear steps prior to safe implementation of machine learning in clinical practice for cardiovascular diseases and other disease areas.

**Supplementary Information:**

The online version contains supplementary material available at 10.1186/s12916-021-01940-7.

## Background

Disease definitions rely on expert consensus, informed by best available evidence [1–3]. Machine learning (ML), the use of algorithms to describe patterns in datasets with (supervised) or without (unsupervised) the need to define them a priori, is increasingly used in research for definition [4] and risk prediction [5]. However, established evaluation frameworks for clinical utility of such models [6, 7] have not been applied.

Better definitions for cardiovascular disease (CVD), the single greatest disease burden in the UK and globally [6], may improve prevention and treatment by characterising target populations, enabling comparability and generalisability across study designs and populations. Heart failure (HF), acute coronary syndromes (ACS) and atrial fibrillation (AF) are among the commonest CVDs globally [8, 9]. Despite frequent changes in disease definitions [3, 10, 11], continued diagnostic and prognostic uncertainties have led to ML research in all three diseases [12–17]. HF, ACS and AF frequently overlap, so phenotyping and risk prediction are relevant across diseases. To date, no existing ML models are routinely used for CVD management, and their phase of clinical utility is unknown, partly due to a lack of clear criteria.

Unlike drugs and devices [18, 19], the pathway to translation from research to routine use is unclear for ML. The AI-TREE criteria (Additional file_Web table 1) were developed for ML healthcare research [20], but consensus is lacking for best practice or regulatory requirements for implementation [21, 22]. Importance of such guidelines is emphasised by systematic reviews showing that ML performs no better than logistic regression or healthcare professionals in prediction across diseases, with high risk of bias and limited external validation in published studies [23, 24].

Evidence for ML in subtype definition and risk prediction in HF, ACS and AF has not been assimilated. Moreover, it is unclear how application of ML in CVD compares with other diseases. Our aims were to (i) develop a simple framework for clinical utility and validity of ML models in healthcare and (ii) conduct a systematic review to evaluate methods and results of ML for subtype definition or risk prediction in HF, AF and ACS.

## Methods

We followed the Preferred Reporting Items for Systematic reviews and Meta-Analysis (PRISMA) statement, with a protocol agreed by all authors, without prospectively registering.

### Systematic review

#### Identification of studies

We searched PubMed, MEDLINE and Web of Science databases from 1 January 2000 until 31 December 2019. Our search terms, agreed by co-authors, pertained to machine learning, clustering, cardiovascular disease, heart failure, atrial fibrillation, acute coronary syndromes, subtype and risk prediction. Reference lists and expert recommendations were taken into account, to identify grey literature, including conference reports and proceedings, guidelines, working papers and theses (Additional file_Search strategy).

#### Selection of studies

All abstracts were independently screened and then full text of selected abstracts were respectively assessed for eligibility by two reviewers (from AB, SC and MZ), and conflicts were resolved by a third reviewer (AB or SC).

#### Inclusion and exclusion criteria

Studies were eligible if the publication presented:

(i) ML models for disease subtype definition/clustering for HF, ACS and AF; or
(ii) Risk prediction for HF, ACS or AF

Studies were excluded if they:

(i) Were not original empirical data
(ii) Were not English language
(iii) Were not peer-reviewed (e.g. a published dissertation was not eligible)
(iv) Did not concern models developed for humans
(v) Did not have full text available

#### Data extraction

Tools for extraction were adapted and developed with consensus among co-authors from published frameworks for new risk markers (AHA [6]), diagnostic accuracy (QUADAS-2 [25]), prognostic tools (CHARMS [26], PROGRESS [7], TRIPOD [27]) and ML (AI-TREE [20], Christodoulou [23]) (Additional file_Web table 2). The author, year and country of study, clinical setting, data source, outcome, comparator methods, ML method(s) and covariates were extracted. The final checklist was in the three stages of the translational pathway (“development”, “validation” and “impact”) as per AHA and PROGRESS statements [6, 7]. There were seven domains under the three main stages: clinical relevance, patients, algorithms (“development”), internal validation, external validation (“validation”), and clinical utility and effectiveness (“impact”). The questions for each domain were from published guidelines as above with similar extraction items for subtype definition and risk prediction studies. Data extraction was by two independent reviewers (from AB, MZ and SC), and disagreements were resolved by a third reviewer (AB or SC). Quantitative analysis was beyond the scope of this review and its aims.

## Results

### Systematic review of HF, AF and ACS

Of 5918 articles identified by our search, 97 met the inclusion criteria (Additional file_Figure S1).

#### Unsupervised ML for subtype definition

Of the 40 studies of unsupervised ML for subtype definition (included patients: median *n* = 606; min 117, max 251,451), there were 27 in HF, 9 in ACS and 4 in AF. All studies focused on a single disease, and only 6 (15%) studies included data regarding history of all three diseases. Twenty-three (57.5%) studies involved < 1000 individuals (range 117–874) and 29 (72.5%) were based in North America with no analyses from low- or middle-income countries. Across diseases, 26 (65%) studies were in outpatients (8 inpatient and 6 mixed inpatient and outpatient) and 11 used trial data (10 prospective cohort, 5 retrospective cohort, 11 cross-sectional, 5 registries), with 7 using EHR data. The mean number of covariates was 31 (min 3, max 156), most commonly demographic and symptom variables (Table 1) [28–67].

**Table 1:**
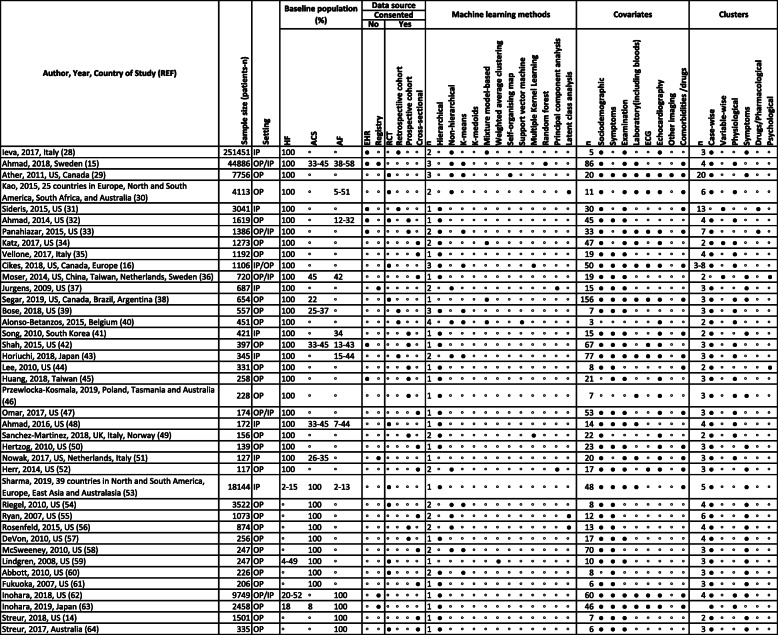
Systematic review of machine learning studies of subtype definition in heart failure, acute coronary syndromes and atrial fibrillation ( *n* = 40 studies)

*ACS*-acute coronary syndrome; *AF*-atrial fibrillation; *CVD*-cardiovascular disease; *ECG*- electrocardiogram; *ED*-emergency department; *EHR*-electronic health records; *HF*-heart failure; *IP*-hospital inpatient; *LV*-left ventricular; *MI*-myocardial infarction; *OP*- hospital outpatient; *RCT*- randomised controlled trial; United Kingdom; *US*-United States.

◦ Negative/No for all columns (except in "Baseline population" column, where it denotes "Unreported")

● Positive/Yes

Most studies used only one ML method (*n* = 22). Non-hierarchical clustering was used in 12 studies (hierarchical: *n* = 25). The most commonly used method was K-means (*n* = 11), but other methods included Gaussian mixture modelling, latent class analysis and random forest (RF). All studies reported disease clusters. Most studies found 3 clusters (*n* = 17, 42.5%; median 3), and most clusters were based on symptom (*n* = 20) or physical (*n* = 17) variables. Clustering was usually case-wise (*n* = 37), rather than variable-wise (*n* = 3) and one study used both approaches.

At the development stage, there were limitations at “clinical relevance” (question related to patient benefit 15.0%, target condition applicability 62.5% and data suitable for clinical question 70%), “patient” (patient applicability 30.0%, low patient selection bias 15.0%) and “algorithm” (algorithm applicability 55.0%, low algorithm bias 40.0%) levels. Twelve studies did not validate or replicate findings; 28 (70.0%) had internal validation (most commonly using number of clusters or prediction of mortality/admissions) and only 5 (12.5%) externally validated findings. There were significant deficiencies under “clinical utility” (improved prediction of outcomes 32.5%, methods available 0%, clinically relevant metrics 72.5%, interpretable by clinicians 65%, clinically justified results 40%) and “effectiveness” (no studies showing effectiveness, real-world or cost) domains (Table 2) [28–67].

**Table 2:**
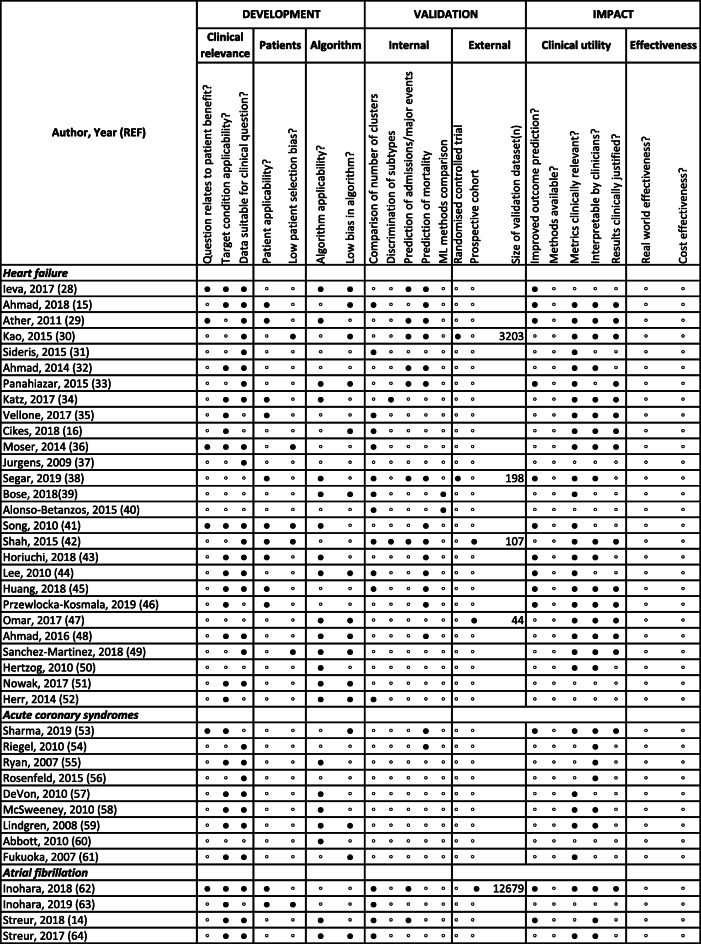
Quality assessment of machine learning studies of subtype definition for heart failure, acute coronary syndromes and atrial fibrillation ( n = 40)

◦ Negative/No

● Positive/Yes

#### Supervised ML for risk prediction

Of the 57 studies of supervised ML for risk prediction (median *n* = 6769; min 28, max 2,994,837), 31 were in HF, 19 in ACS and 7 in AF. Ten of 57 studies involved < 1000 individuals and most were from North America (*n* = 32), with one from a low- or middle-income country (*n* = 1). Risk prediction studies focused on development of (i) HF, ACS or AF in healthy individuals or the general population by case-control, cross-sectional or cohort design (*n* = 25) and (ii) outcomes in HF, ACS or AF (*n* = 32) (Table 3) [13, 45, 53, 65–118].

**Table 3:**
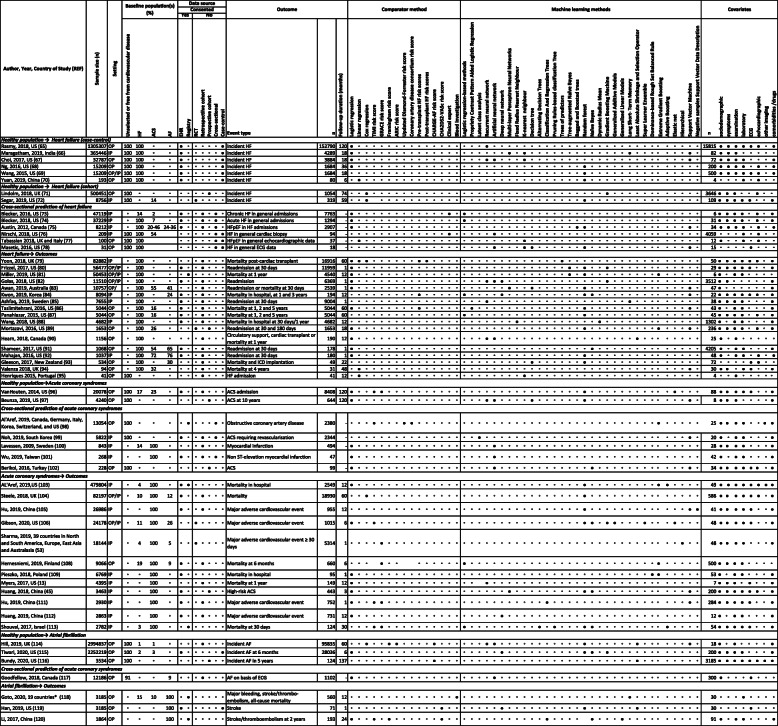
Machine learning risk prediction studies in heart failure, acute coronary syndromes and atrial fibrillation (*n* = 57)

*ACS*, acute coronary syndrome; *AF*, atrial fibrillation; Atherosclerosis Risk in Communities Study; *CHARGE* Cohorts for Heart and Aging Research in Genomic Epidemiology; *CHA2DS2-VASc* congestive heart failure, hypertension, age > 75, diabetes mellitus, stroke, vascular disease, sex category; *CVD*, cardiovascular disease; *ECG*, electrocardiogram; *EHR*, electronic health records; *GRACE*, Global Registry of Acute Coronary Events; *HF*, heart failure; *HFpEF*, heart failure with preserved ejection fraction; *IP*, hospital inpatient; *LV*, left ventricular; *OP*, hospital outpatient; *RCT*, randomised controlled trial; *TIMI*, thrombolysis in myocardial infarction; *UK*, United Kingdom; *US*, United States

*Australia, Austria, Brazil, Canada, China, Denmark, Korea, Finland, France, Germany, Italy, Japan, Mexico, Norway, Poland, Spain, Sweden, Netherlands, and UK

◦ Negative/no for all columns (except in the “Baseline population” column, where it denotes “unreported”)

● Positive/yes

Across diseases, 27 studies were in outpatients (23 inpatient and 7 mixed inpatient and outpatient) and 5 used trial data (6 prospective cohort, 30 retrospective cohort, 6 registry and 11 case-control). Thirty-one studies used EHR data. The mean number of covariates was 723 (median 48; min 6, max 15,815), most commonly demography, symptoms and comorbidities/drugs. The ML methods used were variable with neural networks (*n* = 19), random forest (*n* = 23) and support vector machine (*n* = 16).

At the development stage, there were concerns at “clinical relevance” (question related to patient benefit 78.9%, target condition applicability 68.4% and data suitable for clinical question 68.4%), “patient” (patient applicability 43.9%, low patient selection bias 15.8%) and “algorithm” (algorithm applicability 68.4%, low algorithm bias 47.4%) levels. Only three studies (5.3%) had external validation in the clinical trial (*n* = 12,063), prospective cohort (*n* = 861) and registry (*n* = 4759) data. There was internal validation in 49/57 (86.0%) by “hold-out”, “leave one out” or k-fold cross-validation. The risk of bias was high in all studies with the commonest causes as patient selection (*n* = 47), patient applicability to the clinical question (*n* = 35) and bias in the algorithm(s) (*n* = 31). Again, there were major limitations under “clinical utility” (improved prediction of outcomes 91.2%, methods available 45.6%, clinically relevant metrics 49.1%, interpretable by clinicians 47.4%, clinically justified results 43.9%) and “effectiveness” (no studies showing effectiveness, real-world or cost) domains (Table 4) [13, 45, 53, 65–118].

**Table 4:**
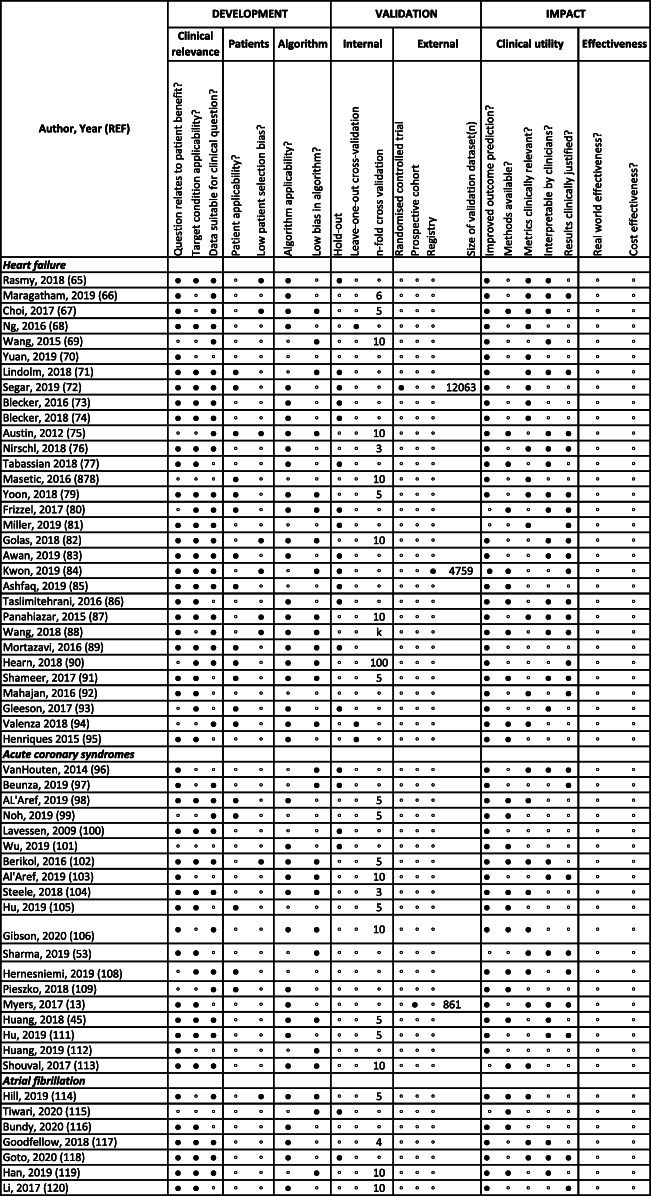
Quality assessment of machine learning studies of risk prediction for heart failure, acute coronary syndromes and atrial fibrillation (n = 57)

◦ Negative/No

● Positive/Yes

## Discussion

Our study highlighted three key findings with important impact on the use of ML in subtype definition and risk prediction for HF, ACS and AF. First, there is significant variation in methods, data and reporting of results. Second, we provide a pragmatic framework, based on published criteria, for assessing validity and clinical utility of ML studies. Third, there are major limitations at development, validation and impact stages in studies of ML in CVD, and our scoping review (Additional file_Scoping review_Methods and Results, Additional file_Web table 3 [119–129], Additional file_Web table 4 [130–143]) [144, 145] suggests similar problems for non-CVDs. Addressing these issues has potential to improve future data science approaches and enhance value of these methods for clinical decision-making and better patient care.

There is increasing interest in guidelines for development and implementation of ML in healthcare from patients and public, academics and industry, partly due to current uncertainty regarding necessary approval and regulatory procedures [146, 147]. Whether in medicines or medical devices, the “development pipeline” (e.g. phases I to IV of drug development) and guidelines are much clearer, ultimately promoting clinical effectiveness and patient safety. The wide variations in methods, datasets and reporting are not surprising, given the current lack of such guidelines with respect to the use of ML in subtype definition and risk prediction [147]. On the basis of existing checklists, we have developed a straightforward 7-domain checklist (16 points) to capture development, validation and impact. Although there are multiple efforts to standardise reporting guidelines for AI in healthcare [27, 148], the relationship with the “translational pathway” in terms of development, validation and impact has not been emphasised. We show that no published studies have results which are ready to be implemented, again likely to be related to lack of consensus regarding what is required in research and clinical practice to ensure clinical effectiveness and safety of ML.

### Problems at the development stage

Studies to date of ML in HF, ACS and AF have been limited by number and type of covariates, population size, geographic location (mainly North American) and clinical setting. As with epidemiologic studies and trials, the generalisability of data and reproducibility of methods [6, 7] are crucial to make findings interpretable and translatable to clinical care. However, the majority of studies to date have not fully considered these factors, resulting in high risk of bias in all studies.

EHR data and advanced data analytics, which have been either under-used or under-reported in studies to date, offer research opportunities across diseases, but ML studies have focused on single diseases, when diseases often co-exist, as for HF, ACS and AF. Outside CVD, perhaps the most promising ML studies to date have been in either large imaging datasets or settings where there is linkage of multimodal clinical data. Whereas the popular concept of ML suggests the use of a wide range of covariates, particularly in clustering, the number and type of variables have been limited. Availability of covariates and quality of recording in EHR determine these limitations rather than research or clinical need. In the future, as more complete and larger EHR datasets become available for research, studies of ML can focus on improved use of limited data in clinical practice, or use of more comprehensive lists of covariates and coexisting diseases for the discovery of new factors in disease definition or prediction. For example, the ESC classification lists 89 causes of HF, which have not been studied together in a single population-based study in EHR or otherwise [3].

The majority of studies have had positive results suggesting publication bias (not formally assessed here), which likely overestimates potential healthcare impact. Lessons must be learned from biomarkers and genomics [149, 150], where lack of standardisation and biased reporting have contributed to lack of translation from science to practice. It is concerning that a significant proportion of studies ML are being developed with questions unrelated to patient benefit, or with data unsuitable to answer them. “Data-driven care” is important in both personalised and precision medicine [151], both of which can benefit from advances in the use of ML. However, a “data-centred” or “data-driven” agenda must remain “patient-centred” rather than “technology-centred” (or in this case, “ML-centred”) [152]. Our framework can be used to plan studies, facilitating clinical relevance and reducing bias.

### Problems at the validation stage

In order to improve data-driven characterisation of CVD and influence clinical decision-making, ML studies for subtyping and prediction should be larger-scale, across diseases, with standardised reporting and proven external validity. To date, the degree of external validation across subtype definition and risk prediction ML studies has been disappointing, making ML difficult to implement in routine care. External validation will help to understand which clustering and prediction tools are of greatest use, and greater availability of electronic health record data should facilitate this step in the pathway to implementation of ML.

### Problems at the impact stage

There are major gaps at the phases of clinical utility, particularly open availability of methods and interpretability of methods and results by clinicians. A limitation of our study is that we did not conduct meta-analysis for studies of either subtype classification or risk prediction because it was out of scope for our review. However, variation and lack of standardisation in methods and reporting make meta-analysis challenging and potentially unrepresentative. These issues can be addressed at design and implementation phases, but have been relatively neglected. Importantly, in applications of ML in CVD to date, studies of effectiveness and cost-effectiveness are lacking. Without these evaluations, ML cannot be implemented safely or effectively.

**Fig. 1:**
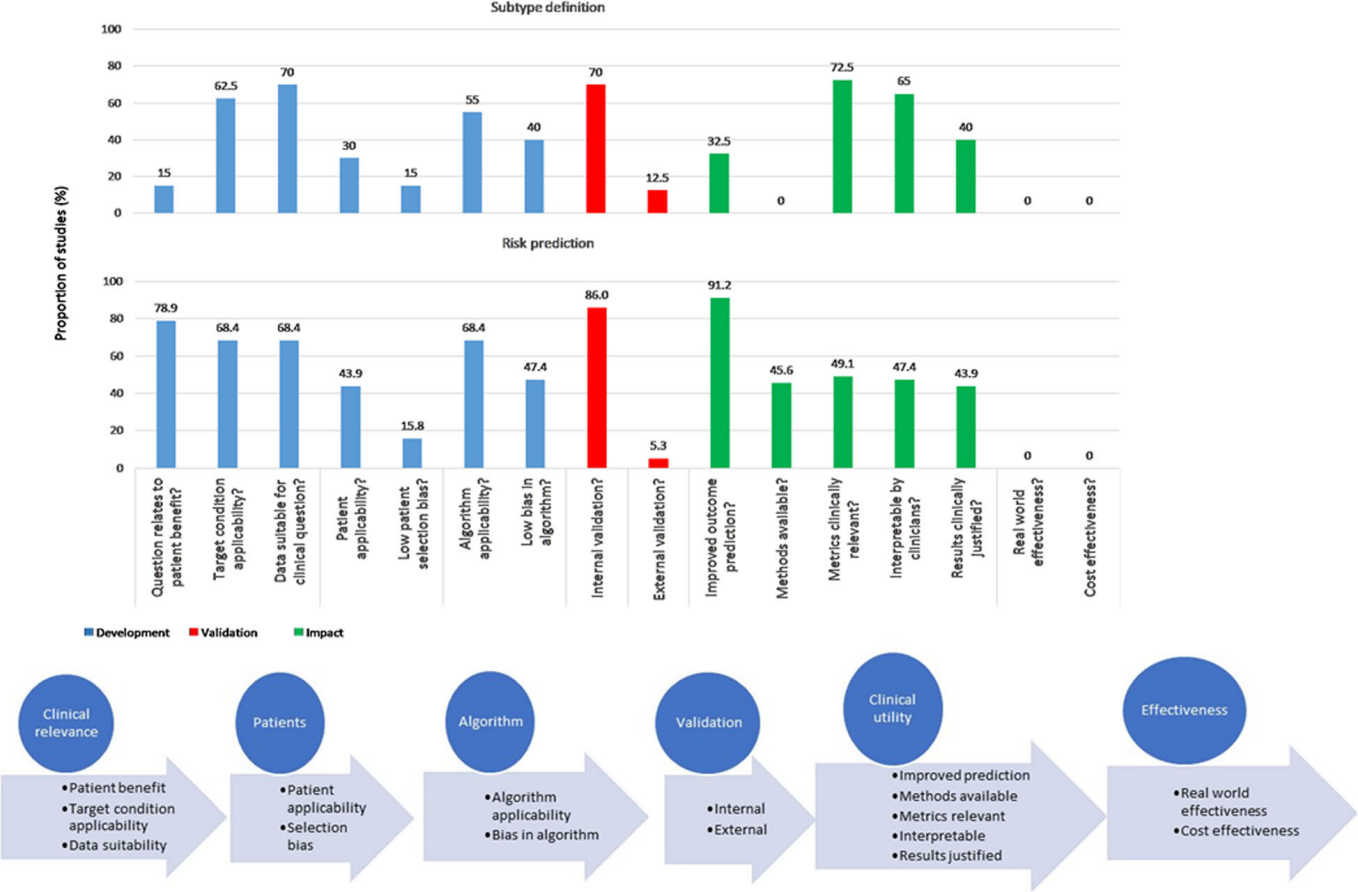
Development, validation and impact of machine learning studies in subtype definition and risk prediction for heart failure, acute coronary syndromes and atrial fibrillation

Figure 1 shows deficiencies at development, validation and impact stages and that ML research is not necessarily being conducted in a “sequential” manner, e.g. to ensure that the evaluation of impact is in validated ML models, perhaps reflecting lack or under-use of consensus guidelines. Particularly in CVD, with high disease burden globally and in low- and middle-income countries, the impact of ML has to be considered through a global lens. The fact that the majority of the literature regarding ML in CVD is from the North American context does raise concern that AI could broaden research and clinical inequalities both within and across countries, beyond current debates about the inequalities which may be inherent in algorithms. If individuals are not represented in the data feeding into the algorithms, then the AI cannot benefit them. These inequalities still exist in other domains, such as pharmaceutical trials and genomics, but the situation is improving, e.g. the proportion of RCTs recruiting in low- to middle-income countries has increased in recent years. As EHR and digital healthcare become global phenomena, there is scope to use ML in diverse data and settings. Validation of ML applications in clustering and risk prediction across countries and settings, even between high-income countries, are urgently needed to advance this agenda. We have proposed steps to improve the development and validation of ML in clinical datasets (Additional file_Web table 5).

## Conclusions

Studies to date of ML in HF, ACS and AF have been limited by number and type of covariates, population size, geographic location (mainly North American), clinical setting, focus on single diseases (where overlap and multimorbidity are common) and ML methods used. Moreover, flaws at stages of development, validation and impact reduce the clinical utility and likelihood of implementation of ML in routine healthcare. To improve the generalisability, applicability and clinical utility of ML in CVD and other diseases, and to influence clinical decision-making internationally, we provide a simple checklist to foster standardised reporting and validation.

## Supplementary Information

**Additional file 1.** Search terms and search strategy.

**Additional file 2 Figure S1.** PRISMA flow diagram.

**Additional file 3 Web Table 1.** AI-TREE checklist.

**Additional file 4 Web Table 2.** Data extraction for included studies.

**Additional file 5.** Methods and Results for scoping review.

**Additional file 6 Web Table 3.** Subtype classification studies in other disease areas.

**Additional file 7 Web Table 4.** Risk prediction studies in other disease areas.

**Additional file 8 Web Table 5.** Proposed methods for development and validation of ML.

**Additional file 9.** PRISMA checklist.

## Data Availability

The data underlying this article are available in the article and in its online supplementary material.
