# Supplementary material for: Machine learning for subtype definition and risk prediction in heart failure, acute coronary syndromes and atrial fibrillation: systematic review of validity and clinical utility
Source: BMC Med. 2021 Apr 6;19:85. doi: 10.1186/s12916-021-01940-7 (PMC8022365; doi:10.1186/s12916-021-01940-7)
Supplement: Supplementary file 1 — Additional file 1. Search terms and search strategy. [file 12916_2021_1940_MOESM1_ESM.docx]

**Search terms**

| **POPULATION** | **INTERVENTION** | **OUTCOME** |
| --- | --- | --- |
| **Heart failure [mh]** | **Machine learning [mh]** | **Phenotype [mh]** |
| **Atrial fibrillation [mh]** | **Cluster analysis [mh]** | **Risk [mh]** |
| **Acute coronary syndrome [mh]** | **Artificial intelligence [mh]** | **Mortality [mh]** |
| **Cardiovascular diseases [mh]** | **Unsupervised [tw]** | **Prognosis [mh]** |
| **Myocardial infarction [mh]** | **Supervised [tw]** | **Risk prediction [tiab]** |
| **Heart failure [tw]** | **Machine learning [tw]** | **Classification [mh]** |
| **Atrial fibrillation [tw]** | **Artificial intelligence [tw]** | **Subtype [tw]** |
| **Acute coronary syndrome [tw]** |  | **Subgroup [tw]** |
| **Myocardial infarction [tw]** |  | **Cluster [tw]** |
| **Cardiovascular [tw]** |  | **Phenotype [tw]** |
|  |  | **Prognosis [tw]** |
|  |  | **Prediction [tw]** |

**mh: MeSH term; tw: text word; tiab: title/abstract**

**Search strategy:** Pubmed

“(Heart failure [mh] OR Atrial fibrillation [mh] OR Acute coronary syndrome [mh] OR Cardiovascular diseases [mh] OR Myocardial infarction [mh] OR Heart failure [tw] OR Atrial fibrillation [tw] OR Acute coronary syndrome [tw] OR Myocardial infarction [tw] OR Cardiovascular [tw]) **AND** (Machine learning [mh] OR Cluster analysis [mh] OR Artificial intelligence [mh] OR Unsupervised [tw] OR Supervised [tw] OR Machine learning [tw] OR Artificial intelligence [tw]) **OR** (Phenotype [mh] OR Risk [mh] OR Mortality [mh] OR Prognosis [mh] OR Risk prediction [tiab] OR Classification [mh] OR Subtype [tw] OR Subgroup [tw] OR Cluster [tw] OR Phenotype [tw] OR Prognosis [tw] Prediction [tw])”

1. (Heart failure [mh] OR Atrial fibrillation [mh] OR Acute coronary syndrome [mh] OR Cardiovascular diseases [mh] OR Myocardial infarction [mh] OR Heart failure [tw] OR Atrial fibrillation [tw] OR Acute coronary syndrome [tw] OR Myocardial infarction [tw] OR Cardiovascular [tw]): 1,561,798

2. Machine learning [mh] OR Cluster analysis [mh] OR Artificial intelligence [mh] OR Unsupervised [tw] OR Supervised [tw] OR Machine learning [tw] OR Artificial intelligence [tw]): 182, 669

3. (Phenotype [mh] OR Risk [mh] OR Mortality [mh] OR Prognosis [mh] OR Risk prediction [tiab] OR Classification [mh] OR Subtype [tw] OR Subgroup [tw] OR Cluster [tw] OR Phenotype [tw] OR Prognosis [tw] Prediction [tw]): 68,567

1 AND 2 OR 3: 78,071

1 AND 2 AND 3: 510
