# Supplementary material for: Machine learning for subtype definition and risk prediction in heart failure, acute coronary syndromes and atrial fibrillation: systematic review of validity and clinical utility
Source: BMC Med. 2021 Apr 6;19:85. doi: 10.1186/s12916-021-01940-7 (PMC8022365; doi:10.1186/s12916-021-01940-7)
Supplement: Supplementary file 2 — Additional file 2 Figure S1. PRISMA flow diagram. [file 12916_2021_1940_MOESM2_ESM.docx]

**Figure S1: PRISMA flow diagram to show included studies**

Publications identified through searching PubMed, MEDLINE, and Web of Science (n=5918)

Additional records identified through reference lists and conference proceedings (n=58)

**Identification**

Records after duplicates removed

(n=2635)

**Screening**

Records screened by title or by title and abstract

(n=2635)

Records excluded

(n=2505)

**Eligibility**

Full-text articles assessed for eligibility

(n=130)

Full-text articles excluded

(n=33)

**Included**

Studies included in review

(n=97)
