# Supplementary material for: Machine learning for subtype definition and risk prediction in heart failure, acute coronary syndromes and atrial fibrillation: systematic review of validity and clinical utility
Source: BMC Med. 2021 Apr 6;19:85. doi: 10.1186/s12916-021-01940-7 (PMC8022365; doi:10.1186/s12916-021-01940-7)
Supplement: Supplementary file 3 — Additional file 3 Web Table 1. AI-TREE checklist. [file 12916_2021_1940_MOESM3_ESM.docx]

**Web table 1: AI-TREE checklist for Health-related ML/AI Technology**

***Overall Project-specific Question***

*1) How is the ML/AI model embedded in feedback loops as part of a learning health system?*

***Inception-specific Questions***

*2) What is the health question relating to patient benefit?*

*3) When and how should patients be involved in data collection, analysis, deployment, and use?*

*4) Is there organisational transparency about the flow of data?*

***Analysis-specific Questions***

*5) Is the data suitable to answer the clinical question, i.e. does it capture the relevant real-world heterogeneity, and is it of sufficient detail and quality?*

*6) Does the methodology reflect the real-world constraints and operational procedures associated with data collection and storage?*

*7) On what basis are data accessible to other researchers?*

*8) What computational and software resources are available, and are they sufficient to tackle this problem?*

*9) Are the reported performance metrics relevant for the clinical context in which the model will be used?*

*10) Is the reported gain in statistical performance with the ML/AI algorithm clinically justified in the context of any trade-offs?*

*11) Is the ML/AI algorithm compared to the current best technology, and against other appropriate baselines?*

*12) Are the different parts of the prediction modelling pipeline available to others to allow for methods reproducibility, including: the statistical code for ‘pre-processing’, and the modelling workflow (including the methods, parameters, random seeds, etc. utilised)?*

*13) Are the results reproducible in settings beyond where the system was developed (i.e. external validity)?*

***Impact Evaluation-specific Questions***

*14) What evidence is there that the model does not create or exacerbate inequities in healthcare by age, sex,ethnicity or other protected characteristics?*

*15) What evidence is there that clinicians and patients find the model and its output (reasonably) interpretable?*

*16) What evidence is there of real world model effectiveness in the proposed clinical setting?*

***Implementation-specific Questions***

*17) Is the model being regularly re-assessed, and updated as data quality and clinical practice changes (i.e.post-deployment monitoring)?*

*18) Is the ML/AI model cost-effective to build, implement, and maintain?*

*19) How will the potential financial benefits be distributed if the ML/AI model is commercialized?*

*20) How have the regulatory requirements for accreditation/approval been addressed?*
