# Supplementary material for: Machine learning for subtype definition and risk prediction in heart failure, acute coronary syndromes and atrial fibrillation: systematic review of validity and clinical utility
Source: BMC Med. 2021 Apr 6;19:85. doi: 10.1186/s12916-021-01940-7 (PMC8022365; doi:10.1186/s12916-021-01940-7)
Supplement: Supplementary file 4 — Additional file 4 Web Table 2. Data extraction for included studies. [file 12916_2021_1940_MOESM4_ESM.docx]

**Web table 2: Items for extraction for development, validation and impact for included machine learning studies of subtype definition and risk prediction**

| **Item for extraction** | **Definition** | **Source** |
| --- | --- | --- |
| **DEVELOPMENT** | | |
| **Clinical relevance** | | |
| Question relates to patient benefit? | Is there a health question relating to patient benefit?  Y/N | AI-TREE |
| Target condition applicability? | Is there concern that the target condition as defined does not match the research question?  Y/N | QUADAS-2 |
| Data suitable for clinical question? | Is the data suitable to answer the clinical question, i.e. does it capture the relevant real-world heterogeneity, and is it of sufficient detail and quality?  Y/N | AI-TREE |
| **Patients** | | |
| Patient applicability? | Is there concern that the included patients do not match the research question? Y/N | QUADAS-2 |
| Patient selection bias? | Could the selection of patients have introduced bias?  Y/N | QUADAS-2 |
| **Algorithm** | | |
| Algorithm applicability? | Is there concern that the algorithm, its conduct, or interpretation differ from the research question?  Y/N | AI-TREE |
| Bias in algorithm? | Could the variable selection, predictor selection or interpretation of the machine learning have introduced bias?  Y/N | Christodoulou, CHARMS |
| **VALIDATION** |  |  |
| **Internal**: | | |
| *Method*  Comparison of number of clusters | Assessment of optimal number of clusters in dataset?  Y/N  Assessment of discrimination of algorithm (e.g. sensitivity, specificity, area under the curve)?  Y/N  Assessment of prediction of or hospital admissions or major events?  Y/N  Assessment of prediction of mortality?  Y/N  Is the ML/AI algorithm compared to the current best technology, and against other appropriate baselines (including different methods of ML)?  Y/N | AI-TREE,  CHARMS,  AHA, PROGRESS, TRIPOD |
| Discrimination of subtypes |  |  |
| Prediction of admissions/major events |  |  |
| Prediction of mortality |  |  |
| ML methods comparison |  |  |
| Hold-out | *Method of internal validation:*  Hold-out= simplest cross-validation where the dataset is split into a 'training' and 'testing' set.  Y/N  Leave-one-out cross-validation=when number of folds equals the number of instances in the data set.  Y/N  N-fold cross-validation=when the train dataset is split into “n” folds.  Y/N | Christodoulou, CHARMS |
| Leave-one-out cross-validation |  |  |
| N-fold cross-validation |  |  |
| **External:** | | |
| Randomised controlled trial | Source and size of external validation dataset | AHA, PROGRESS, TRIPOD |
| Prospective cohort |  |  |
| Registry |  |  |
| Size of validation dataset (n) |  |  |
| **IMPACT** | | |
| **Clinical Utility** | | |
| Improved outcome prediction | Is there evidence of improved risk prediction?  Y/N | AHA, PROGRESS, TRIPOD |
| Methods available? | Are the different parts of the prediction modelling pipeline available to others to allow for methods reproducibility, including: the statistical code for ‘pre-processing’, and the modelling workflow (including the methods, parameters, random seeds, etc. utilised)?  Y/N | AI-TREE |
| Metrics clinically relevant? | Are the reported performance metrics relevant for the clinical context in which the model will be used?  Y/N | AI-TREE |
| Interpretable by clinicians? | Is there evidence that clinicians and patients find the model and its output (reasonably) interpretable?  Y/N | AI-TREE |
| Results clinically justified? | Is the reported gain in statistical performance with the ML/AI algorithm clinically justified in the context of any trade-offs?  Y/N | AI-TREE |
| **EFFECTIVENESS** |  |  |
| Real world effectiveness | Is there evidence of real world model effectiveness in the proposed clinical setting?  Y/N | AI-TREE, AHA |
| Cost effectiveness | Is there evidence of cost effectiveness in the proposed clinical setting?  Y/N | AHA |

**◼ Subtype definition ◼ Risk prediction**
