# Supplementary material for: Machine learning for subtype definition and risk prediction in heart failure, acute coronary syndromes and atrial fibrillation: systematic review of validity and clinical utility
Source: BMC Med. 2021 Apr 6;19:85. doi: 10.1186/s12916-021-01940-7 (PMC8022365; doi:10.1186/s12916-021-01940-7)
Supplement: Supplementary file 5 — Additional file 5. Methods and Results for scoping review. [file 12916_2021_1940_MOESM5_ESM.docx]

**Scoping review of ML for subtype definition or risk prediction in other diseases.**

***Methods***

Scoping reviews do not give detailed answers to specific questions but rather provide an overview of a broad field(W123, W124). Using similar search terms and inclusion/exclusion criteria, we performed a scoping review of ML in disease subtyping and risk prediction for diseases other than HF, AF and ACS.

**Results**

Of 1815 identified studies, 11 studies of subtype definition and 14 studies of prediction were selected for inclusion in our scoping review included.

The studies using clustering approaches were in diseases ranging from Type 2 diabetes and Crohn’s disease to dementia and sepsis. They were mainly in outpatients (n=8; and min 211, max 100000) and from US (n=6), US and Europe (n=1), Sweden (n=1), UK (n=2) and Korea (n=1). Cohorts (n=10) and RCT (n=2) were the main sources of data, with four studies using EHR data. Covariates (min 5, max 6812) were mostly sociodemographic and physical, and the number of clusters was 3-16. Ten studies answered a question relating to patient benefit, 8 studies had low patient selection bias, 3 had low algorithm bias. Four studies externally validated findings in prospective cohorts, and all had internal validation. Two studies reported improved risk prediction, 6 had openly available methods, 5 had clinically relevant metrics, 9 were interpretable by clinicians and 6 had clinically justified results. No studies showed real world- or cost- effectiveness (**Web Table 3**).

The 14 studies of supervised ML for risk prediction were in a spectrum of diseases from breast cancer and acute kidney injury to diabetic retinopathy and haemodialysis. Four of the studies were in healthy individuals for risk of incident CVD. The setting varied from outpatient (n=9) to inpatient (n=2) and both (n=3). Study designs involved cohorts (n=13), case-control (n=2) or registry (n=1) and 7 used EHR data (min 4676, max 703782). Outcomes for prediction included need for referral (retinal disease), deterioration (acute kidney injury, asthma) or mortality and five studies considered onset of diseases (CVD, diabetes, hypertension and multiple comorbidities). Seven studies were from the US, 5 from the UK and the remainder from Zambia, Singapore and Kuwait. Covariates ranged from 4 to 620000, and mainly related to socio-demography, symptoms or comorbidities. Multiple ML methods were frequently compared (n=10), but those with only one ML method were usually testing neural networks. The comparator usually included logistic or Cox regression (n=10) and the two studies of retinal disease compared with expert ophthalmologists. Thirteen studies related to patient benefit, 10 had low patient selection bias, and 7 had low bias in algorithms. All studies had internal validation; one had external validation and a further ongoing study proposed external validation in a published protocol. All studies reported improved outcome prediction. Eight studies showed real-world effectiveness but none reported cost-effectiveness. Only two studies had openly available methods (**Web Table 4**).
