# Supplementary material for: Machine learning for subtype definition and risk prediction in heart failure, acute coronary syndromes and atrial fibrillation: systematic review of validity and clinical utility
Source: BMC Med. 2021 Apr 6;19:85. doi: 10.1186/s12916-021-01940-7 (PMC8022365; doi:10.1186/s12916-021-01940-7)
Supplement: Supplementary file 7 — Additional file 7 Web Table 4. Risk prediction studies in other disease areas. [file 12916_2021_1940_MOESM7_ESM.docx]

**Web Table 4. Machine learning risk prediction studies in other disease areas (n=14)**
